# Patients who take their symptoms less seriously are more likely to have colorectal cancer

**DOI:** 10.1186/1471-230X-12-130

**Published:** 2012-09-22

**Authors:** Barbara-Ann Adelstein, Petra Macaskill, Robin M Turner, Les Irwig

**Affiliations:** 1 Prince of Wales Clinical School, Faculty of Medicine, University of New South Wales, Sydney, Australia; 2 Screening and Test Evaluation Program, School of Public Health, University of Sydney, Sydney, Australia

**Keywords:** Symptom perception, Colorectal cancer, Questionnaire, Reliability, Predictive value of tests

## Abstract

**Background:**

People vary in how they respond to symptoms. The purpose of this study was to assess whether serious disease is more likely to be present in patients who report that they take any symptoms less seriously than other people do, and to assess the reliability of a question which can be used to identify the extent to which patients take any symptom seriously. To do this we assessed whether the likelihood of detecting colorectal cancer is higher in patients who report that they take symptoms less seriously than other people do.

**Methods:**

Cross sectional study of 7736 patients who had colonoscopy to find colorectal cancer. Before colonoscopy, patients completed a questionnaire on bowel symptoms and were also asked: “Compared to other people of your age and sex, how seriously do you think you take any symptoms?” Likelihood of detecting colorectal cancer according to responses to this question was assessed by logistic regression models, unadjusted and adjusted for symptoms and other known predictors of colorectal cancer.

Question reliability was assessed in a different sample using percentage agreement and the kappa statistic for the answers given by each patient on two occasions. Agreement between patient and doctor responses was also assessed (n = 108).

**Results:**

Patients who reported they took symptoms less seriously were 3.28 (95%CI: 2.02, 5.33) times more likely to have colorectal cancer than patients who took symptoms more seriously than others. The effect was smaller (1.85 (95%CI: 1.11, 3.09)), but remained statistically significant in models including symptoms and other predictors of colorectal cancer. The question was reliable: on repeat questioning, 70% of responses were in absolute agreement and 92% were within 1 category, kappa 57%. Patient-doctor agreement was 66%, within 1 category 92%, kappa 48%.

**Conclusion:**

Patients who take their symptoms less seriously have a considerably higher likelihood of colorectal cancer than those who identify themselves as taking any symptoms more seriously than other people. The question is easy to ask and has good reliability. Doctors also reliably identify how patients assess themselves. Assessment of how seriously patients take any symptoms can contribute to the clinical assessment of a patient.

## Background

People vary in how they respond to symptoms. Some may not take symptoms seriously while others may become concerned about even minor symptoms. Individual perceptions about the seriousness of symptoms may affect decisions to consult a health practitioner. People who take symptoms seriously are probably more likely to ask for medical help at a symptom severity which would prompt no action by someone who takes their symptom less seriously.

There is surprisingly little research on the variability in how seriously people take their symptoms or how this is associated with clinical outcomes. Research has explored factors influencing people to seek medical attention [1,2], and it is accepted that patients’ knowledge, beliefs and expectations about an illness determines their appraisal of their symptoms, illness and subsequent health behaviour [3,4]. It has been suggested that patients ability to “detect” their symptoms – to know which symptoms may be potentially important in cancer diagnosis – is associated with seeking medical advice in a timely manner [5,6]. It has been demonstrated that patients cope with adverse events, including experiencing symptoms, differently depending on their coping style [7]. Anxiety [8] and personality type [9] influence people’s decision to seek medical help. Some health seeking behaviour research has been concerned with ‘worry’ which often overlaps with risk perception. Worry has been shown to be associated with test results indicative of pathology. For example, in men undergoing screening for prostate cancer, worry about having the disease was associated with a higher prostate-specific antigen (PSA), even after controlling for other factors such as perceived cancer risk, and cancer–related symptoms [10]. However, there is a dearth of research on whether how seriously a person takes their symptoms is associated with a finding of serious disease, such as colorectal cancer.

Further, while there are several questionnaires that evaluate issues related to how seriously people take symptoms [11], each tests only one specific aspect of a complex issue, and each comprises several questions. There is no single, simple measure. The absence of such a simple reliable tool hinders research about whether patients who take their symptoms seriously present earlier in the course of disease or have less serious underlying illness.

Our aim was to assess whether serious disease is more likely to be present in patients who report that they take any symptoms less seriously than other people do, and to assess the reliability of a question which can be used to identify the extent to which patients take any symptom seriously. These objectives were addressed using data from the CRISP (Colonoscopy Research in Symptom Prediction) study [12,13]. We investigated whether the seriousness with which a patient takes their symptoms is predictive of a finding of colorectal cancer on colonoscopy, both unadjusted and also adjusted for variables that were included in a multivariable prediction model previously developed using CRISP data [12,13].

## Methods

The CRISP study was a cross sectional study of 7736 patients aged over 18 years who were scheduled to undergo colonoscopy (see Adelstein [12] for a detailed description of the methods of this study). Prior to colonoscopy, patients were asked to complete a comprehensive self-administered questionnaire that elicited data relating to their bowel symptoms, socio-demographic characteristics, medical and family history, use of aspirin and other non-steroidal anti-inflammatory medications, and previous colonoscopy. The questionnaire has previously been shown to be repeatable within patient and between patient and doctor [14], and a copy is available electronically via the publication website. Findings at colonoscopy were collated from endoscopic records. All cancers diagnosed were confirmed by histology.

The questionnaire contained an item relating to symptom perception: “Compared to other people of your age and sex, how seriously do you think you take any symptoms?” The five possible answers were: a lot less seriously than other people; less seriously than other people; about the same as other people; more seriously than other people; and a lot more seriously than other people. Methods for assessing whether responses to this question were associated with colorectal cancer, and also the repeatability of the question are outlined below.

### Assessment of predictive validity

In keeping with our published investigation of the association between bowel symptoms and colorectal cancer, these analyses were based on the 159 patients found to have cancer and the 7577 who had no cancer or adenoma.

The distribution of patients across the five categories relating to how seriously patients took their symptoms, and the prevalence of cancer within each category was calculated. The five categories were reduced to three for subsequent analyses by combining “a lot less seriously” with “less seriously” and also combining “much more seriously” with “more seriously”. The relative frequency of responses and the prevalence of cancer for these three categories were computed separately for males and females, according to: age (<50, 50–59, 60–69, 70+ years); anaemia (present vs absent); and the presence and frequency of symptoms (rectal bleeding, rectal mucus and abdominal pain). Informed by our previously published model [12], abdominal pain and mucus symptoms were coded as <12 months duration, occurring weekly; <12 months duration, occurring monthly/occasionally; or none/>12 months duration. Bleeding was coded as <12months duration, occurring weekly; <12 months duration, occurring monthly/occasionally; or no bleeding />12 months duration.

Logistic regression was used to assess the association between how seriously patients took their symptoms and colorectal cancer, both univariately and also adjusted for variables included in the previously developed multivariable model to predict the presence of cancer (age, gender, previous colonoscopy, history of diverticular disease, aspirin and non-steroidal inflammatory drug use, anaemia, rectal bleeding, rectal mucus and abdominal pain) [12]. Although one objective was to assess the incremental gain of adding how seriously patients took their symptoms to the established prediction model, interactions between this variable and age, gender and each of the symptoms listed above were also investigated. The likelihood ratio test was used to assess statistical significance with a two-sided p-value <0.05 used as the criterion for significance.

All statistical analyses were undertaken using SAS statistical software [15].

### Reliability of the question on symptom perception

The reliability of the question relating to symptom perception was evaluated using the same methodology and the same patients as the published evaluation of the reliability of the questions relating to symptoms in the questionnaire [14]. Patient-patient agreement (reproducibility) was assessed in a group of 138 patients who completed the self-administered questionnaire immediately prior to their consultation with the doctor, and who were mailed the same questionnaire to complete an average of 4.2 weeks later (median 3.3 weeks).

Patient–doctor agreement was assessed in another group of 108 patients by comparing the patient’s questionnaire response to how seriously they took their symptoms prior to the consultation with their specialist’s response to the same question based on their perception of the patient during the consultation. The specialist was blinded to the patient’s response.

For both patient-patient and patient-doctor agreement, the proportion of responses showing absolute agreement across all five categories was computed. The weighted kappa statistic (κ), using linear weights, was computed. The McNemar test was used to assess evidence of a systematic direction for disagreements.

### Ethics committee approval

The study received approval from the Ethics Committees of the University of Sydney, Central Sydney Area Health Service (both CRGH and Central Zones), Northern Sydney and Central Coast, Western Sydney Area Health Service and the Sydney Adventist Hospital.

## Results

**Figure 1:**
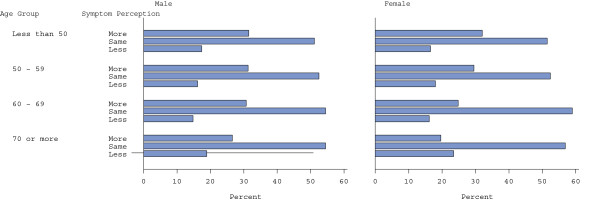
Age-specific percent of patients who report that they took any symptoms more, the same or less seriously than others.

Of the 7,736 patients in the study, only 102 did not answer the question relating to symptom perception and were excluded from further analysis. About half reported that they took their symptoms with the same seriousness as other people. Of the remaining patients, those who said they took their symptoms more seriously were almost twice as numerous as those who said took their symptoms less seriously (Table 1). The pattern of responses was similar for males and females: 53.1% of males and 54.6% of females reported taking their symptoms as seriously as others, 30.1% of males and 27.3% of females took their symptoms more seriously, and 16.8% of males and 18.2% of females took the symptoms less seriously than others. Increasing age was accompanied by a smaller percentage of people who reported taking symptoms more seriously than others, especially in females (p for trend p < 0.001 for females and p = 0.03 for males; Figure 1 and detailed table in Additional file 1: Table S1).

**Table 1:** The distribution of symptom perception and the cancer prevalence, for all five symptom perception categories

How seriously patients thought they took any symptoms*	**Total**		**Cancer**		Unadjusted OR (95% CI)**
	**N**	**%**	**Cases**	**Prevalence%**	
A lot less seriously	362	4.7	10	2.8	1.38 (0.71, 2.68)
Less seriously	976	12.8	39	4.0	2.02 (1.37, 2.98)
Same [Referent Group]	4,115	53.9	83	2.0	1.00
More seriously	1,799	23.6	22	1.2	0.60 (0.38, 0.97)
A lot more seriously	382	5.0	3	0.8	0.39 (0.12, 1.22)
Total	7634	100	157		

*Self assessment, compared to others of the same age.

**p < 0.001; p-value for trend <0.0001, p-value for departures from linear trend = 0.056).

**Figure 2:**
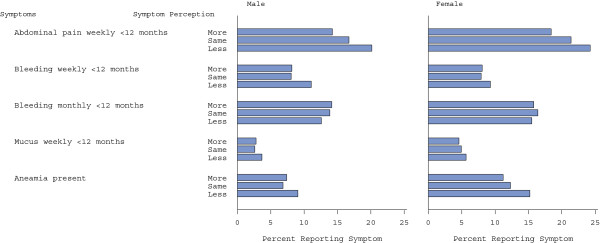
Percentage of patients reporting symptoms, by whether they take any symptoms more, the same or less seriously than others.

The percentage of patients reporting abdominal pain decreased as the seriousness with which patients took their symptoms increased (Figure 2); this trend was evident for both men (p for trend =0.002) and women (p for trend =0.002). For men, the percentage reporting abdominal pain decreased from 20.1% in those who took their symptoms less seriously to 14.2% in those who took symptoms more seriously; the corresponding percentages for females are 24.3% and 18.4%. The percentage of women reporting anaemia also decreased as the seriousness with which they took their symptoms increased (Figure 2, p for trend = 0.014) but no such trend was evident for males (p = 0. 2). There was no evidence of association between how seriously patients took their symptoms and how likely they were to report rectal bleeding or mucus. Details are given in Additional file 2: Table S2.

**Figure 3:**
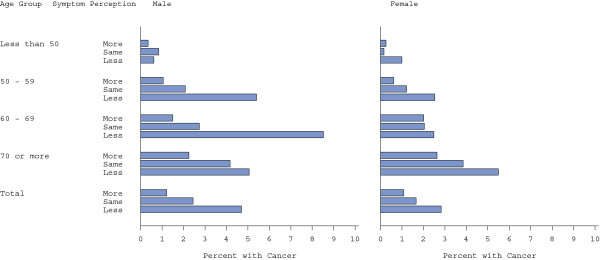
Effect of age and gender on cancer rate in each symptoms perception group.

How seriously patients reported they took any symptoms was associated with colorectal cancer (Table 1). After collapsing the responses into three categories and using those who reported taking any symptoms with the same seriousness as others as the referent group, those who reported that they took any symptoms less seriously had almost a 2-fold higher prevalence of colorectal cancer (OR 1.85, 95%CI: 1.29, 2.64) compared to the referent group, whereas those who reported taking any symptoms more seriously had about half the prevalence (OR 0.56, 95%CI: 0.36, 0.88) compared with the referent group (Table 2). Hence, those who reported taking their symptoms less seriously were 3.28 (=1.85/0.56) (95%CI: 2.02, 5.33) times more likely to have cancer than those to who took their symptoms more seriously. Overall, the cancer prevalence in males was inversely related to how seriously they took their symptoms relative to others: 1.2%, 2.4%, and 4.7% corresponding to “more seriously”, “same” and “less seriously”. For females, the corresponding cancer prevalences were 1.1%, 1.7% and 2.8%. The prevalence gradient was evident in almost all age groups (Figure 3, and details are given in Additional file 3: Table S3). Although the gradient seemed strongest in men in their 50s and 60s, this was easily explained by chance with p = 0.57 for the interaction term for age group by sex by how seriously patients took any symptoms in the multivariable model.

**Table 2:** Unadjusted and adjusted odds of cancer for the three groupings of symptom perception

How seriously patients took any symptoms*	**Unadjusted OR (95% CI)**	**Adjusted OR (95% CI)#**
Less/lot less seriously	1.85 (1.29, 2.64)	1.47 (1.00, 2.14)
Same [Referent Group]	1.00	1.00
More/lot more seriously	0.56 (0.36, 0.88)	0.79 (0.50, 1.27)
P-value	<0.001**	0.04

# Adjusted for age, sex, previous colonoscopy, NSAID use, aspirin use, anaemia, diverticulitis, abdominal pain, bleeding and mucus.

** p-value for trend <0.0001, p-value for departures from linear trend = 0.242.

**Figure 4:**
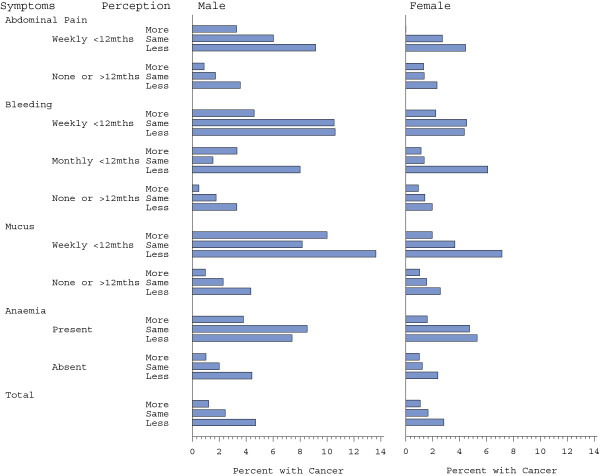
Cancer rate for symptom in each symptom perception group.

Based on the data from this study, we have previously developed a multivariable prediction model for the presence of cancer based on sociodemographic variables (age, gender), medical history (previous colonoscopy, history of diverticular disease, use of aspirin or non-steroidal anti-inflammatory medication) and symptoms (rectal bleeding, rectal mucus, abdominal pain, and anaemia) [12]. The model was demonstrated to have good discrimination with an area under the Receiver Operating Characteristic curve of 0.85 [7]. For these symptoms, the prevalence is higher if the symptom occurs frequently and has been present for less than 12 months. Adding the three category covariate for how seriously patients took any symptoms to this model showed that patients’ perception of their symptoms remained significantly associated with colorectal cancer (full model results are presented in Table 3). The odds ratio gradient was less dramatic after adjustment (Table 2, p = 0.04). After adjustment, patients who took their symptoms less seriously had an odds ratio of 1.47 (95%CI: 1.00, 2.14) compared to the unadjusted estimate of 1.85. Contrasting patients who take their symptoms less serious than others with patients who take their symptoms more seriously than others, the adjusted odds ratio was 1.85 (95%CI: 1.11, 3.09) compared with the unadjusted estimate of 3.28. There was no statistical evidence of interaction between symptom perception and any of the symptoms included in the model. The prevalence of cancer was highest in those patients who took their symptoms less seriously than others for people both with and without each symptom (Figure 4 and Table 4).

**Table 3:** Multivariable model results

**Variables**		**Odds ratio (95% CI)**	**Likelihood ratio P-value**
Previous colonoscopy within last 10 years	No (referent group)		
	Yes	0.24 (0.16, 0.35)	<0.001
Age Group	< 50 years (referent group)	1.00	<0.001
	50 – 59 years	7.09 (3.44, 14.61)	
	60 – 69 years	15.02 (7.36, 30.64)	
	70 years or more	25.10 (12.28, 51.31)	
Anaemia	No (referent group)	1.00	
	Yes	3.59 (2.40, 5.38)	<0.001
NSAID use	No (referent group)	1.00	
	Yes	0.35 (0.16, 0.76)	0.002
Aspirin use	No (referent group)	1.00	
	Yes	0.58 (0.37, 0.93)	0.017
Diverticulitis	None (ref group)	1.00	
	Yes	0.38 (0.17, 0.85)	0.008
Sex	Male (referent group)	1.00	
	Female	0.68 (0.48, 0.95)	0.023
Abdominal Pain	None or occur less frequently than weekly or present > 12 months (referent group)	1.00	
	Occurs weekly; and present <12 months	2.28 (1.60, 3.26)	<0.001
Bleeding	None or present >12 months (referent group)	1.00	
	Occurs monthly/occasionally; and present <12 months	2.07 (1.33, 3.24)	<0.001
	Occurs weekly and present <12 months	5.56 (3.63, 8.53)	
Mucus	None or occur less frequently than weekly or present > 12 months (referent group)	1.00	
	Occurs weekly and present <12 months	2.65 (1.49, 4.73)	0.002
How seriously take symptoms	Less seriously	1.47 (1.00, 2.14)	0.044
	Same (referent group)	1.00	
	More seriously	0.79 (0.50, 1.27)	

NSAID = non-steroidal anti-inflammatory drug.

**Table 4:** Prevalence of cancer within groups defined by the seriousness with which patients take their symptoms, for males and females separately

	**Males**									**Females**								
	**Less**			**Same**			**More**			**Less**			**Same**			**More**		
	Cancer	%	Total	Cancer	%	Total	Cancer	%	Total	Cancer	%	Total	Cancer	%	Total	Cancer	%	Total
**Abdominal Pain**																		
Occurring weekly and present <12 months	11	9.2	120	19	6.0	315	5	3.3	152	8	4.4	180	13	2.7	477	0	0.0	205
Everything else	17	3.6	476	27	1.7	1568	8	0.9	915	13	2.3	562	24	1.4	1755	12	1.3	909
**Bleeding**																		
Occurring weekly and present <12 months	7	10.6	66	16	10.5	152	4	4.6	87	3	4.3	69	8	4.5	177	2	2.2	90
Occurring monthly/ occasionally and present <12 months	6	8.0	75	4	1.5	261	5	3.3	151	7	6.1	115	5	1.4	366	2	1.1	176
Everything else	15	3.3	455	26	1.8	1470	4	0.5	829	11	2.0	558	24	1.4	1689	8	0.9	848
**Mucus**																		
Occurring weekly and present <12 months	3	13.6	22	4	8.2	49	3	10.0	30	3	7.1	42	4	3.6	110	1	2.0	51
Everything else	25	4.4	574	42	2.3	1834	10	1.0	1037	18	2.6	700	33	1.6	2122	11	1.0	1063
**Anaemia**																		
Present	4	7.4	54	4	7.4	54	3	3.8	79	6	5.3	113	13	4.7	275	2	1.6	125
Absent	24	4.4	542	35	2.0	1754	10	1.0	988	15	2.4	629	24	1.2	1957	10	1.0	989
Total	28	4.7	596	46	2.4	1883	13	1.2	1067	21	2.8	742	37	1.7	2232	12	1.1	1114

### Reliability of the question on how seriously people thought they took any symptom

In the patient-patient comparison based on the original 5-category responses, there was complete agreement in 70% and agreement within 1 category in 92% of patients (Table 5). Kappa was 57% (95% CI 43-65%), indicating substantial agreement [16]. There was no significant difference in the direction of the disagreements between the first and second patient completion of the question (McNemar’s test: p = 0.54).

**Table 5:** Patient-patient responses to symptom question (% of total shown, n = 138)

	**Patient second questionnaire**						
Patient first questionnaire		A lot less	Less	Same	More	Much more	Total
	A lot less	1	1	2	1	0	5
	Less	1	8	2	2	0	13
	Same	1	3	41	6	0	51
	More	1	0	4	15	3	22
	Much more	1	0	0	2	5	8
	Total	5	12	49	26	8	100%

In the patient-doctor comparison, there was complete agreement in 66% and agreement within 1 category in 92% of responses (Table 6). Kappa was 48% (95% CI 34-61%), indicating moderate agreement [16]. For the disagreements, there was no systematic effect in the direction of the responses between doctors and patients (McNemar’s test: p = 0.87).

**Table 6:** Patient-doctor responses to symptom question (% of total shown, n = 108)

	**Doctor questionnaire**						
Patient questionnaire		A lot less	Less	Same	More	Much more	Total
	A lot less	0	4	2	0	1	7
	Less	0	6	2	2	0	10
	Same	0	5	45	4	0	55
	More	0	1	6	12	3	22
	Much more	0	0	3	2	2	7
	Total	0	17	58	20	6	100%

## Discussion

In our study, patients who take any symptoms less seriously than others have an almost 2-fold higher prevalence of cancer (OR 1.85, 95%CI: 1.29, 2.64) than those who take their symptoms with the same seriousness as others, and a more than a 3-fold higher prevalence of cancer (OR = 3.28, 95%CI: 2.02, 5.33) than those who take their symptoms more seriously than others. Even if adjustment is made for a wide range of other predictors of colorectal cancer, the effects are still almost 1.5 and 2-fold respectively. An increase in prevalence is apparent in all age groups, in males and females and also in people with, and without, symptoms. The assessment of how seriously people took their symptoms was based on a question which is reliable and easy to answer which only a very small percent of patients did not complete.

Strengths of our study are the use of a reliable question to assess how seriously people reported they took symptoms and testing predictive validity in a large high quality study of an important clinical disease. The question is asked in a general way and is not restricted to any particular set of symptoms or disease. High quality of the predictive validity study was assured by several features. The data were collected prospectively with the question completed before disease was identified, all patients underwent colonoscopy with a caecal intubation rate of 98% and all lesions were examined for pathology.

The weakness of our study is that we investigated only one condition – colorectal cancer – so we cannot be certain that the results would be as striking for other clinical outcomes. That can be readily assessed by researchers adding the question we used to their studies.

We are not aware of any previous tool that encapsulates how seriously patients take their symptom in a single question. It is possible that the question relates to stoicism, on which there is some research. Murray et al. showed that the Liverpool Stoicisim Scale has adequate reliability. However, this requires a 20-item questionnaire. They found that stoicism was associated with lower reported quality of life though they did not explore whether serious clinical diagnoses are more common in stoical people. More generally, they point out that there is very little research on stoicism [17]. Miller has shown that individual differences in health–seeking behaviour and health status are influenced by whether people typically scan for threat-level information (high monitors) or ignore threat-relevant information (low monitors) [7]. High monitors, who are assessed by their doctors as having less severe medical problems, complain about these as much as low monitors. However, measurement of whether people are high or low monitors is by completion of 32 responses to 4 imaginary, stress-invoking scenarios [18]. These measures differ from ours in concept, in that they have been developed for use in people already labelled as having a disease and in their complexity.

We have shown that our simple single question can predict disease, prior to diagnosis, and irrespective of the presence of symptoms. Our single question does not measure or evaluate the underlying constructs and mechanisms that may explain why a person takes their symptoms as seriously as they do; the question effectively provides an assessment of how seriously a person takes their symptoms, and links this to patient outcomes. We are not aware of any other studies that have used a single question in this way.

Our results are based on a clinical population – all patients had been referred for specialist consultation and subsequently underwent colonoscopy, although the question was answered prior to this. The presence of bowel symptoms is a common reason for seeking medical attention [19]. In our study 6 of the symptoms were reported as present by more than 30% of patients. Our cancer detection rate is 1.9%, suggesting it is not a high-risk referred population. Although the population is not highly selected, we think that our finding is not a feature of a general population, but rather reflects who seeks medical help.

One way of explaining our findings is by considering a hypothetical scenario loosely based on our results. Imagine that the colorectal cancer prevalence is about 3% in people going for colonoscopy who report taking symptoms less seriously than others; one can represent this as 3 cancers in 100 people. It seems likely that people who take their symptoms more seriously than others access health services for minor symptoms that do not reflect organic pathology. If they are also referred for colonoscopy, this might add an extra 100 people to the pool of patients investigated, without adding any more cancers, so that the cancer prevalence is now only 1.5%.

Another way of explaining our findings is that people who take their symptoms more seriously present for colonoscopy earlier or more frequently than those who take their symptoms less seriously, allowing polyps to be detected and removed preventing their progression to colorectal cancer.

One might expect the effect on outcomes of how seriously people take their symptoms to disappear once the effect of symptom perception is added to a model containing predictive bowel symptoms. The fact that the gradient of risk for our symptom perception question is diluted but still evident suggests that the effect must be partly mediated by factors outside those measured which are important in people’s health-seeking behaviour.

## Conclusion

Our study has shown that the question presented here is a reproducible tool to assess how seriously a patient takes any symptoms and that the assessment of how seriously patients take symptoms is a useful addition to the armamentarium used by doctors as part of the clinical assessment of patients. Our study needs to be repeated in other populations and with other disease outcomes. If the result is replicable, it suggests that doctors would be wise to have a heightened appreciation that in those who take symptoms less seriously than others, there may be increased risk of serious pathology.

## Competing interests

The authors declare that they have no competing interests.

## Authors’ contributions

BA and LI conceived and designed the study. BA managed the study and data collection; RT and PM did the statistical analysis; All authors contributed to the interpretation of the data, and ideas. BA and LI led the drafting of the paper, but all authors contributed significantly. LI is the guarantor of the study and made the final decision to submit. All authors had full access to the data and take responsibility for the integrity of the data and the accuracy of the data analysis, and for the final version published.

## Pre-publication history

The pre-publication history for this paper can be accessed here:

http://www.biomedcentral.com/1471-230X/12/130/prepub

## Supplementary Material

Additional file 1 **Table S1.** Distribution of how seriously people take their symptoms by age group, for males and females**.** Table showing number (and percentage) of males and females in each of the age groups <50, 50-59, 60-69 and >70 years shown by whether they take their symptoms less, the same as or more seriously than others. Click here for file

Additional file 2 **Table S2.** Distribution of the severity of symptoms within groups defined by the seriousness with which patients take their symptoms, for males and females separately. Table showing number (and percentage) of males and females, grouped by severity of each of abdominal pain, rectal bleeding, much and anaemia, shown by whether they take their symptoms less, the same as or more seriously than others. Click here for file

Additional file 3 **Table S3.** Prevalence of cancer within age groups defined by the seriousness with which patients take their symptoms, for males and females separately.Table showing number (and percentage) of males and females with colorectal cancer in each of the age groups <50, 50-59, 60-69 and >70 years shown by whether they take their symptoms less, the same as or more seriously than others. Click here for file
