# Supplementary material for: Patients who take their symptoms less seriously are more likely to have colorectal cancer
Source: BMC Gastroenterol. 2012 Sep 22;12:130. doi: 10.1186/1471-230X-12-130 (PMC3522996; doi:10.1186/1471-230X-12-130)
Supplement: Additional file 1 — Table S1. Distribution of how seriously people take their symptoms by age group, for males and females. Table showing number (and percentage) of males and females in each of the age groups <50, 50-59, 60-69 and >70 years shown by whether they take their symptoms less, the same as or more seriously than others. [file 1471-230X-12-130-S1.pdf]

Additional file:

Table 1: Distribution of how seriously people take their symptoms by age group, for males and females.

	Male								Female							
	Less		Same		More		Total		Less		Same		More		Total	
	N	%	N	%	N	%	N	%*	N	%	N	%	N	%	N	%**
Age Group (years)																
Less than 50	161	17.4	473	51.1	291	31.5	925	*43.2*	201	16.5	627	51.5	390	32	1218	*56.8*
50 – 59	148	16.2	480	52.5	286	31.3	914	*45.5*	198	18	575	52.4	324	29.5	1097	*54.5*
60 – 69	129	14.8	475	54.5	268	30.7	872	*46.6*	161	16.1	589	59	248	24.8	998	*53.4*
70 or more	158	18.9	455	54.5	222	26.6	835	*51.9*	182	23.5	441	56.9	152	19.6	775	*48.1*
Total	596	16.8	1883	53.1	1067	30.1	3546	*46.5*	742	18.2	2232	54.6	1114	27.3	4088	*53.5*

Note: Less, same and more refer to the answer to the question "compared to other people of your age and sex, how seriously do you take your symptoms?"Less = a lot less seriously + less seriously than others; more = more than and much more than others.

% = percentage in that age group reported separately for males and females

%* = percentage of all patients in that age group who are male; %** = percentage of all patients in that age group who are female;
