# Supplementary material for: Patients who take their symptoms less seriously are more likely to have colorectal cancer
Source: BMC Gastroenterol. 2012 Sep 22;12:130. doi: 10.1186/1471-230X-12-130 (PMC3522996; doi:10.1186/1471-230X-12-130)
Supplement: Additional file 2 — Table S2. Distribution of the severity of symptoms within groups defined by the seriousness with which patients take their symptoms, for males and females separately. Table showing number (and percentage) of males and females, grouped by severity of each of abdominal pain, rectal bleeding, much and anaemia, shown by whether they take their symptoms less, the same as or more seriously than others. [file 1471-230X-12-130-S2.pdf]

Additional File:

Table 2: Distribution of the severity of symptoms within groups defined by the seriousness with which patients take their symptoms, for males and females separately.

	Male								Female							
	Less		Same		More		Total		Less		Same		More		Total	
	N	%	N	%	N	%	N	%	N	%	N	%	N	%	N	%
**Abdominal Pain**																
Occurring weekly and present <12 months	120	20.1	315	16.7	152	14.2	587	16.6	180	24.3	477	21.4	205	18.4	862	21.1
Everything else	476	79.9	1568	83.3	915	85.8	2959	83.4	562	75.7	1755	78.6	909	81.6	3226	78.9
**Bleeding**																
Occurring weekly and present <12 months	66	11.1	152	8.1	87	8.2	305	8.6	69	9.3	177	7.9	90	8.1	336	8.2
Occurring monthly/ occasionally and present <12 months	75	12.6	261	13.9	151	14.2	487	13.7	115	15.5	366	16.4	176	15.8	657	16.1
Everything else	455	76.3	1470	78.1	829	77.7	2754	77.7	558	75.2	1689	75.7	848	76.1	3095	75.7
**Mucus**																
Occurring weekly and present <12 months	22	3.7	49	2.6	30	2.8	101	2.8	42	5.7	110	4.9	51	4.6	203	5.0
Everything else	574	96.3	1834	97.4	1037	97.2	3445	97.2	700	94.3	2122	95.1	1063	95.4	3885	95.0
**Anaemia**																
Present	54	9.1	129	6.9	79	7.4	262	7.4	113	15.2	275	12.3	125	11.2	513	12.5
Absent	542	90.9	1754	93.1	988	92.6	3284	92.6	629	84.8	1957	87.7	989	88.8	3575	87.5
**Total**	596	100.0	1883	100.0	1067	100.0	3546	100.0	742	100.0	2232	100.0	1114	100.0	4088	100.0

Note: there were 0 females who had frequent abdominal pain who took their symptoms more seriously than others
