# Supplementary material for: Patients who take their symptoms less seriously are more likely to have colorectal cancer
Source: BMC Gastroenterol. 2012 Sep 22;12:130. doi: 10.1186/1471-230X-12-130 (PMC3522996; doi:10.1186/1471-230X-12-130)
Supplement: Additional file 3 — Table S3. Prevalence of cancer within age groups defined by the seriousness with which patients take their symptoms, for males and females separately.Table showing number (and percentage) of males and females with colorectal cancer in each of the age groups <50, 50-59, 60-69 and >70 years shown by whether they take their symptoms less, the same as or more seriously than others. [file 1471-230X-12-130-S3.pdf]

Additional File:

Table 3: Prevalence of cancer within age groups defined by the seriousness with which patients take their symptoms, for males and females separately.

	Male									Female								
	Less			Same			More			Less			Same			More		
	Cancer	%	Total	Cancer	%	Total	Cancer	%	Total	Cancer	%	Total	Cancer	%	Total	Cancer	%	Total
Age Group Less than 50	1	0.6	161	4	0.8	473	1	0.3	291	2	1.0	201	1	0.2	627	1	0.3	390
50 - 59	8	5.4	148	10	2.1	480	3	1.0	286	5	2.5	198	7	1.2	575	2	0.6	324
60 - 69	11	8.5	129	13	2.7	475	4	1.5	268	4	2.5	161	12	2	589	5	2.0	248
70 or more	8	5.1	158	19	4.2	455	5	2.3	222	10	5.5	182	17	3.9	441	4	2.6	152
Total	28	4.7	596	46	2.4	1883	13	1.2	1067	21	2.8	742	37	1.7	2232	12	1.1	1114
